# Hydrogen Sulfide Maintained the Good Appearance and Nutrition in Post-harvest Tomato Fruits by Antagonizing the Effect of Ethylene

**DOI:** 10.3389/fpls.2020.00584

**Published:** 2020-05-14

**Authors:** Gai-Fang Yao, Chuang Li, Ke-Ke Sun, Jun Tang, Zhong-Qin Huang, Feng Yang, Guan-Gen Huang, Lan-Ying Hu, Peng Jin, Kang-Di Hu, Hua Zhang

**Affiliations:** ^1^School of Food and Biological Engineering, Hefei University of Technology, Hefei, China; ^2^Xuzhou Institute of Agricultural Sciences of the Xuhuai District of Jiangsu Province, Xuzhou, China; ^3^Department of Ecology and Environment of Anhui Province, Hefei, China

**Keywords:** hydrogen sulfide, ethylene, tomato fruits, nutritional quality, post-harvest ripening

## Abstract

Hydrogen sulfide (H_2_S) could act as a versatile signaling molecule in delaying fruit ripening and senescence. Ethylene (C_2_H_4_) also plays a key role in climacteric fruit ripening, but little attention has been given to its interaction with H_2_S in modulating fruit ripening and senescence. To study the role of H_2_S treatment on the fruit quality and nutrient metabolism, tomato fruits at white mature stage were treated with ethylene and ethylene plus H_2_S. By comparing to C_2_H_4_ treatment, we found that additional H_2_S significantly delayed the color change of tomato fruit, and maintained higher chlorophyll and lower flavonoids during storage. Moreover, H_2_S could inhibit the activity of protease, maintained higher levels of nutritional-related metabolites, such as anthocyanin, starch, soluble protein, ascorbic acid by comparing to C_2_H_4_ treatment. Gene expression analysis showed that additional H_2_S attenuated the expression of beta-amylase encoding gene *BAM3*, UDP-glycosyltransferase encoding genes, ethylene-responsive transcription factor *ERF003* and *DOF22*. Furthermore, principal component analysis suggested that starch, titratable acids, and ascorbic acid were important factors for affecting the tomato storage quality, and the correlation analysis further showed that H_2_S affected pigments metabolism and the transformation of macromolecular to small molecular metabolites. These results showed that additional H_2_S could maintain the better appearance and nutritional quality than C_2_H_4_ treatment alone, and prolong the storage period of post-harvest tomato fruits.

## Introduction

Ripening of fleshy fruit is composed of a series of complex and coordinated processes that leading to edible fruit with desirable flavor (Karlova et al., 2014). However, fruit excessive ripening will cause senescence and deterioration during storage, which finally leads to shortened shelf life and reduction in commercial value (Lee et al., 2010). In this process, fruit ripening is accompanied by profound physiological and transcriptome changes, such as cell wall component degradation, accumulation of sugar and reduction of organic acids, increased volatile compounds, degradation of chlorophyll and accumulation of pigments (Klee and Giovannoni, 2011; Gapper et al., 2013). Ethylene is a gaseous phytohormone that regulates the whole life cycle of plants, including plant growth and development, fruit ripening and organ senescence, plant biotic and abiotic stress, and extensively investigated in a large number of crops (Dubois et al., 2018). Fleshy fruits could be classified as climacteric and non-climacteric which is defined by whether an increase in respiration and concomitant increase in ethylene biosynthesis happened after the start of ripening (Klee and Giovannoni, 2011). In climacteric fruit like tomato, ripening is initiated by ethylene and exogenous ethylene could also promote fruit ripening (Liu et al., 2015). Ethylene coordinates the ripening process and acts in concert with other phytohormones. Chen et al. (2019) reported that exposure to ethylene improved sucrose accumulation in ripening sugarcane. In addition, carotenoid accumulation is modulated by the auxin-ethylene balance during tomato fruit ripening; strawberry fruit ripening was regulated by ABA, IAA, and ethylene (Su et al., 2015; Guo et al., 2018).

Hydrogen sulfide (H_2_S) is a small gasotransmitter that plays an important role in diverse plant physiological processes such as plant adaptation to stress conditions, stomatal movement, root development, autophagy and flower senescence (Hancock and Whiteman, 2016). Accumulating evidence reported that H_2_S delayed senescence in post-harvest fruits and vegetables, including apple, kiwifruit, broccoli, and strawberry, etc. (Zhang et al., 2011; Hu et al., 2012; Li et al., 2014; Zhu et al., 2014; Zheng et al., 2016). Previous researches reported that H_2_S maintains higher metabolites levels in broccoli and strawberry, such as carotenoids, anthocyanin, ascorbic acid, reducing sugars and soluble proteins (Hu et al., 2012; Li et al., 2014). During kiwifruit storage, H_2_S played an important role in delaying kiwifruit ripening and senescence and maintaining higher level of titratable acid and ascorbic acid by repression of ethylene production (Zhu et al., 2014). Meanwhile, H_2_S could protect lipid from peroxidation by improving antioxidative enzyme activities and decreasing the accumulation of reactive oxygen species (ROS) (Zhu et al., 2014). All the evidence showed that ethylene and H_2_S regulate climacteric fruit ripening and senescence, whereas the interaction of H_2_S with ethylene in controlling the post-harvest fruit ripening and the nutritional quality are rarely reported.

Tomato (*Solanum lycopersicum*) is one of the most studied fleshy fruit, and it has been assumed as a “functional food” because of the evidence regarding to the reduced risk of cancer and cardiovascular diseases in relationship to its consumption (Giovannetti et al., 2012). Their benefits to human health are primarily associated with the bioactive compounds in fruit such as lycopene, ascorbic acid, tocopherols and polyphenols, carotenoids, anthocyanins (Giovannetti et al., 2012; Vinha et al., 2014). However, tomato is not resistant to storage and prone to rot, thus resulting in economic loss.

In our recent studies, H_2_S was found to alleviate tomato fruit ripening by reducing ROS accumulation, and meanwhile ethylene biosynthesis and signaling pathway were inhibited by H_2_S (Yao et al., 2018; Hu et al., 2019). Though H_2_S has been found to be a senescence regulator in diverse fruits and vegetables, the mechanism of exogenous H_2_S in affecting the bioactive compounds of ethylene-induced tomato fruit ripening and senescence is still obscure. In the present research, we tried to explore the effect of H_2_S on the mentalism of the bioactive compounds in C_2_H_4_-induced tomato fruit ripening by comparing to C_2_H_4_ treatment alone. Tomato fruits of the cultivar “Micro Tom” at white mature stage were fumigated with exogenous C_2_H_4_ or C_2_H_4_ plus H_2_S, and the bioactive compounds and the expressions of genes involving in nutrient metabolism were investigated during post-harvest storage. Furthermore, PCA, the correlation and the change patterns among the bioactive compounds were analyzed to explore the underlying physiological mechanism.

## Materials and Methods

### Plant Materials and Treatment

Tomato fruits of the cultivar “Micro Tom” were harvested in the man-made glasshouse of School of Food and Biological Engineering, Hefei University of Technology, Anhui province, China. Fruit without pest or mechanical damage were harvested at white mature stage. Random eight fruits were used as one group, and each experiment was composed of three groups. NaHS (purchased from Sigma) was used as the donor of H_2_S and ethephon in aqueous solution for C_2_H_4_ donor. C_2_H_4_ treatment was proceeded with a 100 mL of 1.0 g/L ethephon aqueous solution. C_2_H_4_ + H_2_S co-treatment was composed of a 150 mL of 0.90 mmol⋅L^–1^ NaHS aqueous solution and a 100 mL of 1.0 g/L ethephon aqueous solution. The above samples were placed in 3 L sealed containers. Tomato fruits were fumigated by C_2_H_4_ or C_2_H_4_ + H_2_S for 24 h and the fruits were fumigated by equal amount of distil water. The tomato flesh (without seeds) were sampled every day till 7 days after storage and frozen in liquid N_2_ quickly and stored in −80°C refrigerator.

### Determination of Color Change of Tomato Fruits

Color change of tomato fruits was measured by a colorimeter (model WSC-100, Japan) as shown in Hu et al. (2012) with some modification. Each tomato fruit at the equatorial part was selected for the determination, and the value of *a^∗^/b^∗^* showed color change of fruit surface.

### Determination of Chlorophyll and Carotenoid Contents

Chlorophyll and carotenoid contents of tomato fruits were assayed according to the method of Wellburn (1994). 2.0 ± 0.01 g of fresh tomato sample was ground and extracted by ethanol and 80% acetone solution in a ratio of 1:1 (v/v). The absorbance was measured at 663, 645, and 440 nm. Three replicates were performed for each sample and the results were expressed as mg/g FW (fresh weight).

### Determination of Flavonoids, Total Phenols, and Anthocyanin Contents

The flavonoids contents were determined by aluminum chloride colorimetric assay by measuring the absorbance at 510 nm (Li et al., 2014). Rutin was used as the standard for calibration.

Total phenols were assayed following the method of Pirie and Mullins (1976). The total phenols in fruit was determined by spectrophotometer at 280 nm. Gallic acid was used as the standard to make a calibration curve.

Anthocyanin content was extracted and determined according to the protocol described by Lee and Wicker (1991). 2.0 ± 0.01 g of tomato fruits were ground with 10 mL of 0.1% HCl-methanol solution. The absorbance was recorded at 530, 620, and 650 nm and anthocyanin content were expressed as mg/g FW.

### Determination of Ascorbic Acid, Titratable Acids, and Reducing Sugar Content

Ascorbic acid was assessed by the indophenol titration method with minor changes (Nath et al., 2011). 5.00 ± 0.01 g of fruit sample was homogenized with 5 mL of 2% oxalic acid followed by centrifugation at 12000 g for 30 min. The supernatant was adjusted to 25 mL with 2% oxalic acid and titrated with 2,6-dichlorophenol-indophenol to a pink color and maintain a pink color in 30 s.

The titratable acid content was determined by the method adapted from Bureau et al. (2009). 5.00 ± 0.01 g of tomato homogenates were mixed with 20 mL distilled water. Then, mixture was used for acid-base neutralization to measure the titratable acid content. The volume of NaOH was recorded to calculate the content of titratable acids.

The content of reducing sugar was assessed according to the method of Miller (1959) with some modifications. 2.00 ± 0.01 g of tomato samples were ground with 5 mL of 0.1M Na-acetate buffer with the enzyme solution (both solutions were preheated at 50°C for 5 min). The absorbance at 540 nm was recorded to measure the content of reducing sugar with glucose as the standard to make a calibration curve. Three replicates were performed for each sample and the results were expressed as mg/g FW (fresh weight).

### Determination of the Contents of Soluble Protein and Starch

The content of soluble protein was assayed following the method of Bradford (1976). Absorption of soluble protein was recorded at 595 nm. The content of starch in tomato fruits was determined referring to the method by Zhang et al. (2012). 2.0 g of tomato fruits was homogenized with 3 mL of 80% ethanol solution. The content of starch was determined at 510 nm, and soluble starch was used as the standard for calibration.

### Determination of Proteolytic Enzyme and Amylase Activity

For the activity of protease, tomato flesh (2.0 ± 0.01 g) were ground in 5 mL of ice-cold Tris-HCl buffer [50 mM, pH 7.5, included 1 mM EDTA, 15 mM β-mercaptoethanol, and 1% polyvinyl pyrrolidone (PVP)]. The homogenate was centrifuged and then the supernatant was collected for protease activity determination (Reimerdes and Klostermeyer, 1976). Absorbance was recorded at 540 nm, and the activity of protease was quantified as U/g FW.

The activity of amylase was determined according to the method of Staden and Mulaudzi (2000) with some modifications. 2.00 ± 0.01 g of tomato samples were homogenized with 3 mL of citric acid buffer (pH 5.6, 0.1 mM). The homogenate was centrifuged at 12000 *g* for 20 min at 4°C, and the supernatant was placed in a new tube for amylase determination. One unit of enzyme activity was defined as the amount of maltose produced per gram of the sample per min, and the activity of amylase was quantified as U/g FW.

### Quantitative Reverse Transcription PCR Analysis

Total RNA from 0.1 g frozen tomato fruit samples was extracted by RNA Extraction Kit (Tiangen, Beijing, China). Then cDNA was synthesized by reverse transcription kit (PrimeScript RT Master Mix, Takara, Kyoto, Japan) and further used for quantitative PCR. The specific primers used for qPCR were listed in Supplementary Table S1. The expression of tubulin gene in control tomato was used for the normalization of data.

### Statistical Analysis

Statistical analysis was performed using *t*-test in SPSS 22.0. PCA analysis was performed using factor analysis in dimension reduction, and the rotation method was performed by Varimax with Kaiser Normalization. The correlation analysis and the heatmap analysis were performed by Rstudio software. All data are expressed as means ± standard deviations of the values obtained by three independent measurements.

## Results

### Effect of C_2_H_4_ and H_2_S-C_2_H_4_ Treatment on Tomato Color Change During Post-harvest Storage

**FIGURE 1:**
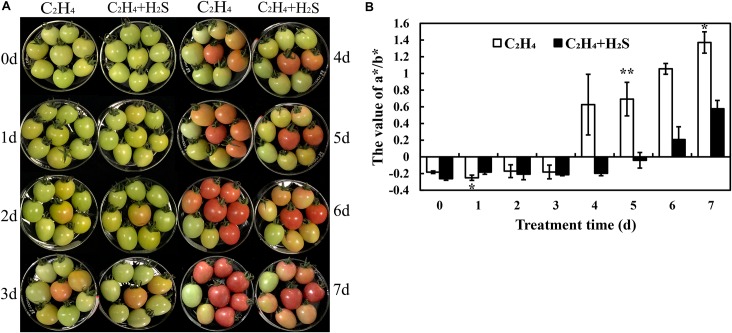
The phenotypic change of post-harvest tomato fruits with C_2_H_4_ or C_2_H_4_-H_2_S treatment. **(A)** The phenotypic change of post-harvest tomato fruits. **(B)** The color parameter a*/b* value changes with C_2_H_4_ or C_2_H_4_-H_2_S treatment during tomato post-harvest storage. a* value represents a range from magenta to green, and b* value represents a range from yellow to blue. Values are the means ± SD (*n* = 3). The experiments and following ones were carried out at room temperature and 85-90% relative humidity. The symbols * and ** stand for significant difference between C_2_H_4_ and C_2_H_4_-H_2_S at *p* < 0.05 and *p* < 0.01, respectively.

The color change of tomato fruit during the post-harvest storage period was shown in Figure 1A. It is clearly showed that all the tomato fruits are at green–yellow (G–Y, ∼30% yellow skin) period on the 0th day. A part of C_2_H_4_-induced tomatoes is at green–orange (G–O, ∼50% orange skin) period on the 1st and 2nd day, but tomato fruits are almost at G–Y period by C_2_H_4_ + H_2_S co-treatment. All C_2_H_4_-induced tomatoes turn to orange–red (O–R, > 90% orange or red skin) from the day 3 to day 5. Meanwhile, some tomatoes are still at G–O period in C_2_H_4_-H_2_S co-treatment. On days 6 and 7, all C_2_H_4_-induced tomato fruits are at the light red (L-R, fully orange or red skin) period, but a part of tomatoes are still at O–R period in C_2_H_4_-H_2_S co-treatment.

As shown in Figure 1B, the change of color was expressed with a^∗^/b^∗^ value. During the whole storage period, the a^∗^/b^∗^ value of the two treatment groups showed an upward trend, while the a^∗^/b^∗^ value of the H_2_S plus ethylene treatment group remained at a lower level compared with ethylene treatment alone. Thus, additional H_2_S treatment can slow the change of tomato color and delay the ripening of tomato fruit during post-harvest storage.

### H_2_S Regulated the Metabolism of Chlorophyll, Anthocyanin, Flavonoids, Carotenoid, Total Phenols in Ethylene-Treated Tomato Fruits

**FIGURE 2:**
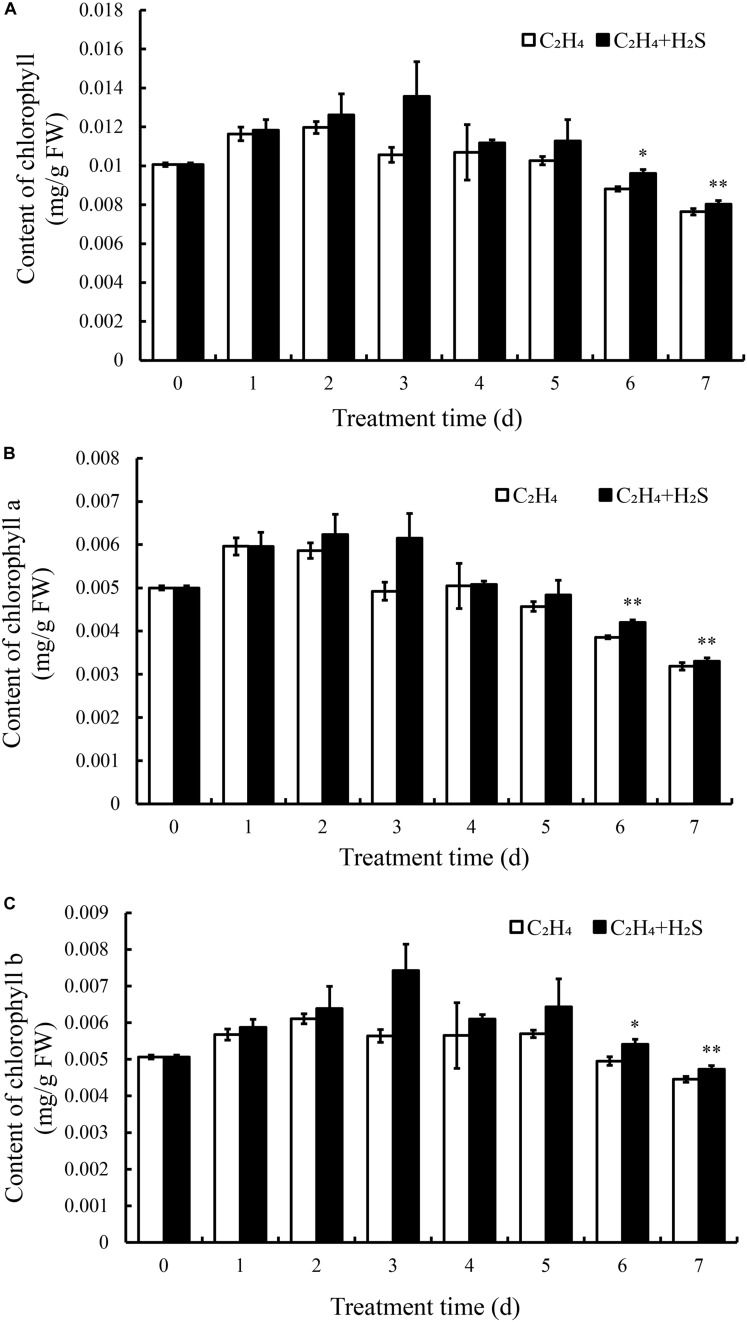
Effects of C_2_H_4_ and C_2_H_4_-H_2_S on the contents of total chlorophyll **(A)**, chlorophyll a **(B)**, chlorophyll b **(C)** in post-harvest tomato. Data are presented as means ± SD (*n* = 3). * and ** in this figure and following ones stand for a significant difference between C_2_H_4_ treatment and C_2_H_4_-H_2_S co-treatment at *p* < 0.05 and *p* < 0.01, respectively.

To further understand the mechanism of H_2_S in alleviating color change of tomato fruits, we determined the contents of chlorophyll, anthocyanin, flavonoids, carotenoid, and total phenols. Total chlorophyll content was composed of chlorophyll a and chlorophyll b. As shown in Figure 2A, chlorophyll content in C_2_H_4_ fumigated tomato increased and reached the peak at the 2st day followed by a gradual decrease until 7th DAS. In contrast, chlorophyll increased and reached the highest level at 3 DAS with C_2_H_4_-H_2_S treatment. Thus, chlorophyll sustained a higher level with C_2_H_4_-H_2_S co-treatment than those of C_2_H_4_ treatment and reached a significant difference on 6th and 7th day (*p* < 0.01 or *p* < 0.05). Meanwhile, the change trend of chlorophyll a and chlorophyll b was the similar to total chlorophyll (Figures 2B,C). The contents of chlorophyll a and chlorophyll b in C_2_H_4_ treatment always sustained a significantly lower level than that of C_2_H_4_-H_2_S co-treatment at 6 and 7 DAS (*p* < 0.01 or *p* < 0.05).

**FIGURE 3:**
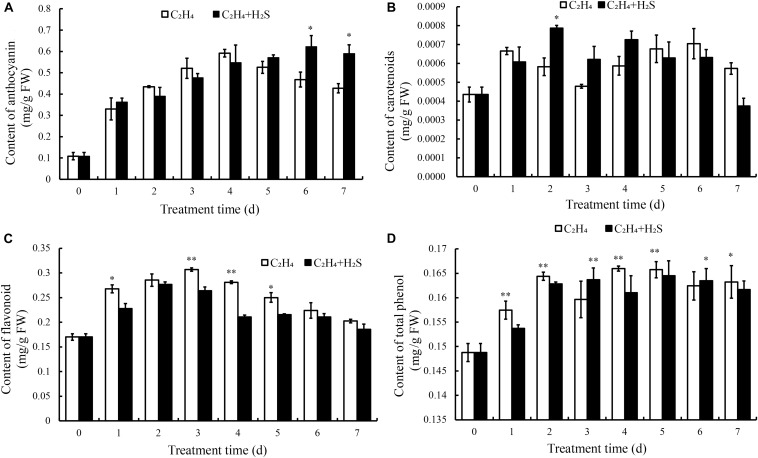
Effect of C_2_H_4_ and C_2_H_4_-H_2_S on the contents of anthocyanin **(A)**, flavonoids **(B)**, carotenoid **(C)**, total phenols **(D)**. Data are presented as means ± SD (*n* = 3). The symbols * and ** stand for significant difference between C_2_H_4_ and C_2_H_4_-H_2_S at *p* < 0.05 and *p* < 0.01, respectively.

Figure 3A shows that anthocyanins increased and peaked at 4 DAS followed by a rapid decrease in C_2_H_4_ treatment, while in C_2_H_4_-H_2_S co-treatment, anthocyanins increased steadily in the first 4 days and remained stable on the day 6 and day 7, and sustained a significantly higher level of anthocyanin at 6 and 7 DAS (*p* < 0.05).

Figure 3B showed the change of carotenoid content in tomato fruit during storage. The carotenoid content was increased firstly, then decreased and then increased with the extension of storage period in C_2_H_4_-H_2_S and C_2_H_4_ treatment. Comparing with C_2_H_4_ treatment, carotenoid content remained at a higher level from day 2 to day 4 in C_2_H_4_-H_2_S co-treatment.

As shown in Figure 3C, flavonoids increased gradually in both C_2_H_4_ and C_2_H_4_-H_2_S treatments until 4 DAS followed by a decrease. The content of flavonoids in the C_2_H_4_ treatment increased and reached the highest level on the day 3, then began to decrease until 7 DAS. The content of flavonoids in the C_2_H_4_-H_2_S treatment decreased from the 2nd day, and was significantly lower than the C_2_H_4_ treatment at 3 and 4 DAS (*p* < 0.01).

In general, Figure 3D showed that total phenols increased steadily during the early storage time. It increased at first, but decreased at 3 DAS, then gradually increased and then tended to be stable in C_2_H_4_ treatment. Similar trends were observed in C_2_H_4_-H_2_S co-treatment, but decreased at 4 DAS.

### Changes of Amylase Activity, Protease Activity, Contents of Starch and Soluble Protein Between C_2_H_4_-H_2_S and C_2_H_4_ Treatment

**FIGURE 4:**
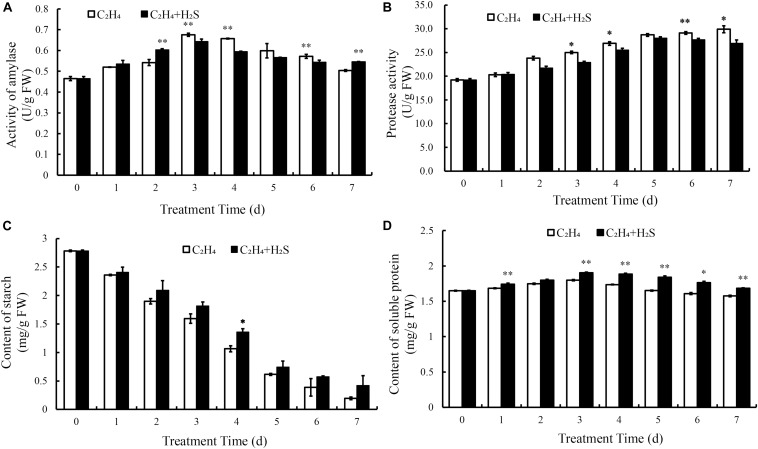
Effect of C_2_H_4_ and C_2_H_4_-H_2_S on activity of amylase **(A)**, protease activity **(B)**, content of starch **(C)** and content of soluble protein **(D)**. Data are presented as means ± SD (*n* = 3). The symbols * and ** stand for significant difference between C_2_H_4_ and C_2_H_4_-H_2_S at *p* < 0.05 and *p* < 0.01, respectively.

To compare the effects of H_2_S treatment on C_2_H_4_-induced tomato fruit quality, amylase activity, protease activity, the content of starch and soluble protein were measured. Figure 4A showed the changes of amylase activity in tomato fruit. It increased and reached the highest level at 3 DAS and then decreased with the storage time in C_2_H_4_ and C_2_H_4_-H_2_S co-treatment. As shown in Figure 4B, protease activity was steadily increased in both treatments during the storage. C_2_H_4_-H_2_S co-treatment maintained significant lower level of protease activity from 3 to 7 DAS except day 5 compared with C_2_H_4_ treatment.

The change of starch content was shown in Figure 4C. In general, with the extension of storage period, the starch content decreased rapidly. The content of starch in C_2_H_4_-H_2_S co-treatment decreased relatively slowly, and maintained a significantly higher level on 4 DAS (*p* < 0.05). The change of soluble protein was shown in Figure 4D. The content of soluble protein increased in the first 3 days of the two treatments. Compared with the C_2_H_4_ treatment, C_2_H_4_-H_2_S co-treatment sustained a significantly higher level of soluble protein content from day 3 to day 7 (*p* < 0.01 or *p* < 0.05), suggesting that H_2_S could alleviate the degradation of protein in tomato fruit during storage.

### Effect of H_2_S on the Contents of Reducing Sugar, Titratable Acid (TA) and Ascorbic Acid, the Ratio of the Reducing Sugar to Titratable Acids

**FIGURE 5:**
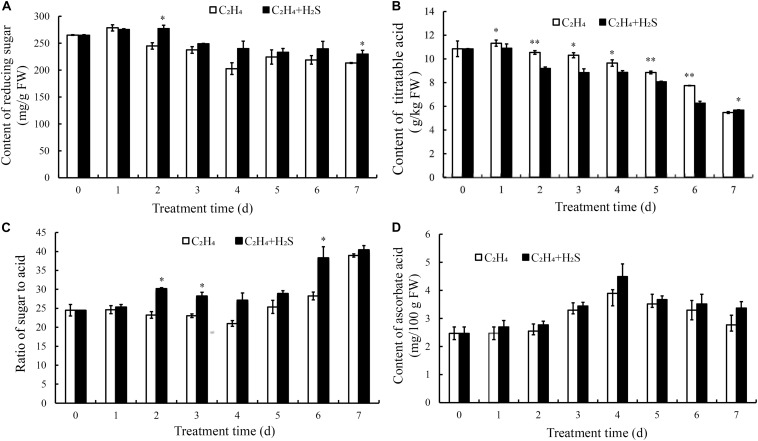
Changes of contents of reducing sugar **(A)**, titratable acid **(B)**, the ratio of sugar to acid **(C)** and ascorbic acid **(D)** in C_2_H_4_ treatment and C_2_H_4_-H_2_S co-treatment. Data are presented as means ± SD (*n* = 3). The symbols * and ** stand for significant difference between C_2_H_4_ and C_2_H_4_-H_2_S at *p* < 0.05 and *p* < 0.01, respectively.

The change of reducing sugar in tomato fruit is shown in Figure 5A. The contents of reducing sugar showed a downward trend in the two treatment groups. Reducing sugar contents in the C_2_H_4_-H_2_S co-treatment had higher level from 2 to 7 days than that those of C_2_H_4_ treatment. Figure 5B showed that TA content decreased gradually in the two treatments. The C_2_H_4_-H_2_S co-treatment sustained a significantly lower level of TA than C_2_H_4_ treatment from 1 to 6 days (*p* < 0.01 or *p* < 0.05).

The sweetness and acidity of the fruit are the important indicators for evaluating the flavor. As shown in Figure 5C, in C_2_H_4_ treatment, the ratio decreased slightly till 4 DAS, then increased gradually. Meanwhile, the ratio sustained a higher level in the C_2_H_4_-H_2_S co-treatment on 2, 3, 6 DAS.

Ascorbic acid is an important nutrient for tomato fruits. As shown in Figure 5D, ascorbic acid contents in both treatments increased gradually and peaked on 4 DAS followed by a decrease. However, there is no significant differences between the two treatments.

### Role of H_2_S on the Transcription of Metabolism and Ripening Related Genes

**FIGURE 6:**
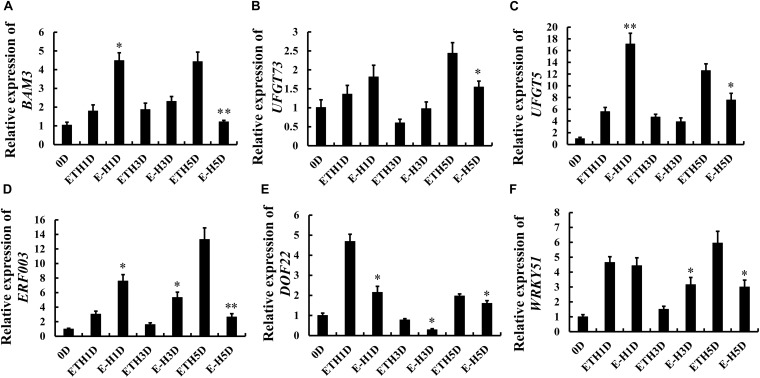
Changes in the gene expression of beta-amylase encoding gene *BAM3* **(A)**, *UFGT73* **(B)**, *UFGT5* **(C)**, ethylene response factor *ERF003* **(D)**, *DOF22* **(E)**, *WRKY51* **(F)** in tomato fruit during storage after C_2_H_4_ and C_2_H_4_ + H_2_S treatment for 1 day. Error bars indicate standard error (*n* = 3). Asterisks indicate significant differences between C_2_H_4_ (ETH) and C_2_H_4_ + H_2_S (E-H) co-treated fruit according to the Student’s *t*-test (*p < 0.05, **p < 0.01).

As shown in Figure 6A, the transcript level of beta-amylase encoding gene *BAM3* increased gradually till day 5 in ethylene-treated tomatoes, whereas ethylene + H_2_S treatment induced the expression of *BAM3* on day 1 and reduced the expression on day 5. Figures 6B,C showed the changes of UDP glucose: flavonoid-3-O-glucosyltransferase encoding genes *UFGT73* and *UFGT5* during tomato storage, respectively. The expression of *UFGT73* fluctuated and H_2_S induced lower transcript level on day 5. However, H_2_S enhanced the expression of *UFGT5* on day 1 while attenuated the expression on day 5 in comparison to C_2_H_4_ treatment. The transcription of several ripening related transcription factor *ERF003*, *DOF22* and *WRKY51* were also analyzed and shown in Figures 6D–F, respectively. Generally, *ERF003* expression showed an increasing trend during storage in C_2_H_4_ group. The increase of *ERF003* expression on day 5 was significantly inhibited by additional H_2_S treatment, though H_2_S induced a mild increase of *ERF003* expression on days 1 and 3. Besides, C_2_H_4_ treatment induced higher expression of *DOF22* on day 1 followed by a decrease, whereas the increase in *DOF22* expression was significantly attenuated by additional H_2_S treatment. An increase in *WRKY51* expression was observed in both treatments on day 1. Additional H_2_S was found to inhibit the expression of *WRKY51* on day 5 and reverse trend was observed on day 3. Thus, the modulation of metabolism and ripening related gene expression by H_2_S may contribute to changes in nutrient metabolism.

### PCA Analysis, Correlation Analysis, and Heatmap of the Changes in Bioactive Substances in Tomato Fruits

**FIGURE 7:**
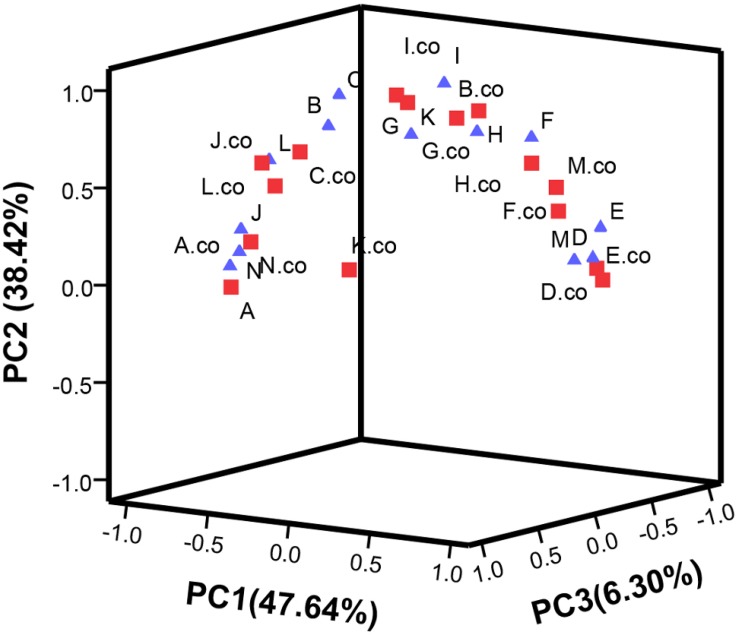
Principal component analysis the main metabolites of post-harvest tomato fruits. PC1∼PC3 were respectively represented the contribution rate of principal components. C_2_H_4_ treatment groups were marked as “co” and square, C_2_H_4_-H_2_S treatment groups were marked by triangle. Metabolites are expressed by A∼N. (A) Protease activity, (B) Soluble protein, (C) Activity of amylase, (D) Starch content, (E) Reducing sugar, (F) Chlorophyll a, (G) Chlorophyll b, (H) Chlorophyll, (I) flavonoid, (J) anthocyanins, (K) Carotenoids, (L) Total phenol, (M) Titratable acid, (N) Ascorbic acid. Data represent the normalized mean values of three independent biological replicates.

Principal component analysis analysis was performed in Table 1 and shown in Figure 7. The contribution rate of PC1, PC2, and PC3 was 47.64, 38.42, and 6.3%, respectively. In PC1, TA, protease activity and starch were the main factors. In PC2, flavonoid, chlorophyll b, and amylase activity were the main factors, while, in PC3, ascorbic acid was the main factor.

**TABLE 1:** The factors score of all the metabolites by principal component analysis in tomato fruits.

Metabolites	Component 1	Component 2	Component 3
Content of titratable acid (C_2_H_4_ + H_2_S)	0.975	0.18	
Protease activity (C_2_H_4_)	–0.95		0.272
Content of chlorophyll b (C_2_H_4_ + H_2_S)	0.948	0.101	–0.25
Content of starch (C_2_H_4_ + H_2_S)	0.939	0.156	–0.228
Content of titratable acid (C_2_H_4_)	0.895	0.427	
Content of Anthocyanidin (C_2_H_4_ + H_2_S)	–0.88	0.191	0.276
Protease activity (C_2_H_4_ + H_2_S)	–0.871		0.376
Content of total phenol (C_2_H_4_ + H_2_S)	–0.806	0.535	0.133
Content of chlorophyll a (C_2_H_4_)	0.796	0.522	–0.109
Content of reducing sugar (C_2_H_4_)	0.783		–0.537
Content of reducing sugar (C_2_H_4_ + H_2_S)	0.776	0.252	–0.529
Content of total phenol (C_2_H_4_)	–0.697	0.427	0.237
Content of chlorophyll (C_2_H_4_)	0.69	0.645	
Content of Anthocyanidin (C_2_H_4_)	–0.633	0.581	0.445
Content of flavonoid (C_2_H_4_ + H_2_S)		0.952	–0.274
Content of flavonoid (C_2_H_4_)		0.95	0.143
Content of chlorophyll b (C_2_H_4_ + H_2_S)		0.93	0.195
Activity of amylase (C_2_H_4_ + H_2_S)	–0.37	0.904	0.15
Content of chlorophyll (C_2_H_4_ + H_2_S)	0.411	0.895	
Content of soluble protein (C_2_H_4_)	0.378	0.876	0.172
Content of soluble protein (C_2_H_4_ + H_2_S)	–0.169	0.83	0.522
Content of chlorophyll b (C_2_H_4_)	0.436	0.798	
Content of carotenoids (C_2_H_4_ + H_2_S)	0.111	0.767	0.191
Content of chlorophyll a (C_2_H_4_ + H_2_S)	0.633	0.761	–0.124
Activity of amylase (C_2_H_4_)	–0.268	0.702	0.631
Content of ascorbic acid (C_2_H_4_)	–0.408	0.259	0.865
Content of ascorbic acid (C_2_H_4_ + H_2_S)	–0.516	0.19	0.81
Content of carotenoids (C_2_H_4_)	–0.39		

**FIGURE 8:**
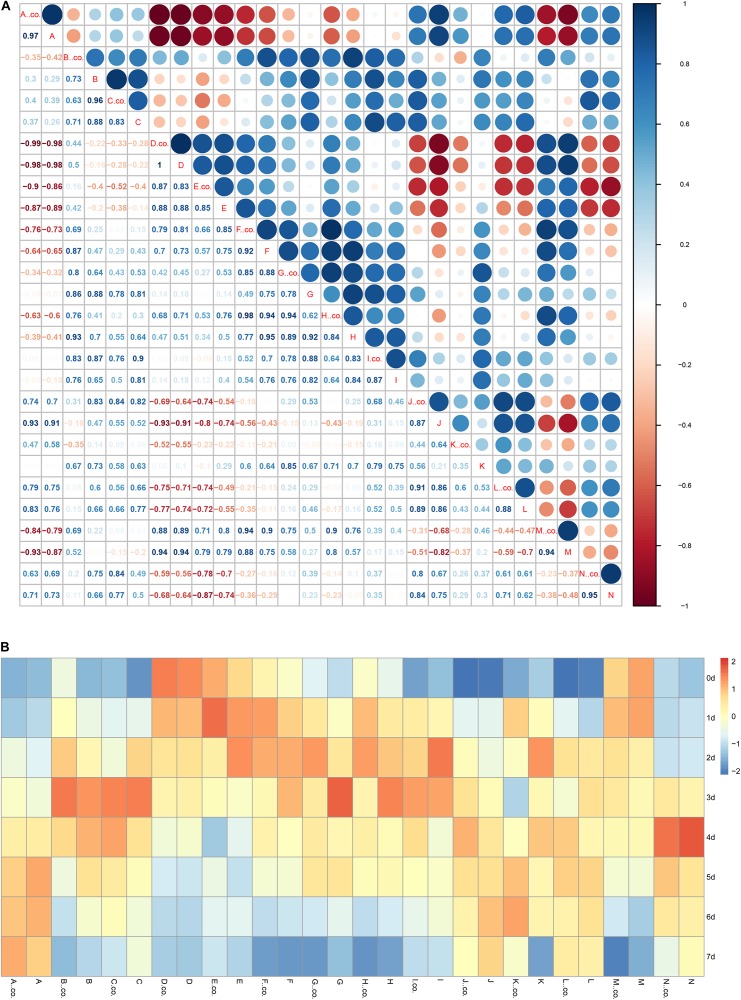
Correlation analysis **(A)** and the heatmap **(B)** among the parameters of bioactive compounds. **(A)** The correlation analysis the relationship among bioactive compounds. Correlation coefficient was analyzed using R scripts. **(B)** The heatmap analyzed the change of bioactive compounds in the storage periods of tomato fruit. co- indicated control group (C_2_H_4_), co-treatment group no marked. (A) Protease activity, (B) Soluble protein, (C) Activity of amylase, (D) Starch content, (E) Reducing sugar, (F) Chlorophyll a, (G) Chlorophyll b, (H) Chlorophyll, (I) flavonoid, (J) anthocyanins, (K) Carotenoids, (L) Total phenol, (M) Titratable acid, (N) Ascorbic acid.

Correlation analysis of various bioactive substances are shown in Figure 8A. Starch and reducing sugar showed a significant negative correlation with ascorbic acid, total phenols, and anthocyanin. Meanwhile, starch and reducing sugar had a significant positive correlation with titratable acid and chlorophyll. Chlorophyll (including chlorophyll a, chlorophyll b) showed a positive correlation with flavonoids, carotenoids, TA, whereas had a negatively correlation with anthocyanins. From the Figure 8A, it can be clearly seen that carotenoids have significant color differences in the C_2_H_4_ treatment and C_2_H_4_ + H_2_S co-treatment, indicating that under the conditions of ethylene fumigation, H_2_S could significantly enhance correlation of carotenoids with other indicators.

As shown in Figure 8B, in general, protease activity, anthocyanins, total phenols, ascorbic acid were gradually increased with the storage time; starch, reducing sugar, titratable acid were gradually decreased. Content of soluble protein, activity of amylase, contents of chlorophyll b and flavonoids were increased firstly, reached the highest level on day 2 or day 3, and then gradually decreased with the storage time. Comparing with C_2_H_4_ treatment, the content of reducing sugar, content of chlorophyll b, content of chlorophyll reached the highest level in the C_2_H_4_ + H_2_S co-treatment later, indicating that under the conditions of ethylene application, H_2_S could slow the degradation of reducing sugar and chlorophyll.

## Discussion

Ethylene, a gaseous hormone with a simple structure, regulates many processes of plant growth and development, including root hair formation, flowering, the senescence and abscission of fruit and leaf, etc. (Dugardeyn and Van Der Straeten, 2008). In the ripening of climacteric fruits, ethylene biosynthesis system 2 called “autocatalytic synthesis” accounts for massive ethylene production (Bapat et al., 2010). Besides, exogenous application of ethylene will hasten ripening process by initiating a signaling cascade. Researchers have developed different means to slowing down post-ripening process in fleshy fruit by genetic engineering or ethylene inhibitor (Bapat et al., 2010). H_2_S, with the odor of rotten eggs, has been found to participate in varied physiological processes in both animal and plant after the discovery of the physiological roles of nitric oxide (NO) and carbon monoxide (CO) (Rochette and Vergely, 2008). In plants, H_2_S has been revealed as a crucial regulator in multiple physiological processes, including seed germination, root morphogenesis, photosynthesis, and flower senescence (Zhang et al., 2008, 2009, 2010, 2011). Recently, it was reported that H_2_S could induce changes in transcriptome and inhibit ethylene production, delay the ripening and senescence of kiwifruits and maintain higher contents of nutrients during storage (Zhu et al., 2014; Lin et al., 2020). Moreover, H_2_S delayed the loss of chlorophyll and respiration in leafy vegetables by inhibiting ethylene production and action (Ubeed et al., 2017).

In this study, the antagonizing effect of C_2_H_4_ and H_2_S on various bioactive substances were estimated in tomato fruits during the post-harvest storage. Color is an important characteristic that reflects the ripening stage of tomato fruit. It has shown that the color change of fruits is closely related to chlorophyll, carotenoids and anthocyanins. In our experiments, as shown in Figures 2A–C, comparing with C_2_H_4_ treatment, C_2_H_4_-H_2_S co-treatment sustained a higher level of chlorophyll and anthocyanin during storage period, but remained a lower level of flavonoid. The similar phenomenon was reported in broccoli, water spinach as well as other living plants (Li et al., 2014; Hu et al., 2015). Correlation analysis showed that chlorophyll has a positive correlation with flavonoids, carotenoids, however, has a negative correlated with anthocyanins (Figure 8A). The shift of the pigments reflects the tomato ripening stage, which provides a good basis for assessing fruit ripening. However, H_2_S could delay the color changes of tomato and slow the ripening process of tomato fruit. Moreover, H_2_S was closely associated with chlorophyll degradation and anthocyanin biosynthesis. It was reported chlorophyllase (CHL) and pheophytinase (PPH) are required in chlorophyll degradation (Schelbert et al., 2009). Moreover, UDP glucose: flavonoid-3-*O*-glucosyltransferase (UFGT) catalyzes anthocyanidins to glucosylated anthocyanins in litchi during fruit coloration (Zhao et al., 2012). In the present research, we found that H_2_S up-regulated the expression of *UFGT5* in the early stage of storage which may contribute to accumulated anthocyanin in H_2_S-treated tomato fruits. However, the regulating role of H_2_S on CHL/PPH in delaying ripening and senescence still needs further study.

H_2_S can maintain the well-appearance of post-harvest broccoli and meanwhile higher nutrient contents, such as, ascorbic acid, reducing sugar and soluble protein than C_2_H_4_ treatment (Li et al., 2014). In the present study, ascorbic acid, reducing sugar, soluble protein and starch were also determined in tomato during post-harvest storage. As shown in Figures 4C,D, 5A,D, tomato fruit with C_2_H_4_-H_2_S co-treatment could sustain higher level of ascorbic acid, reducing sugar and soluble protein, starch than C_2_H_4_ treatment. In PCA analysis, soluble protein, starch and TA, amylase activity and ascorbic acid were the main factors, which could significantly affect tomato fruit quality during post-harvest storage. Thus, H_2_S can alleviate the decreases in ascorbic acid, reducing sugar, soluble protein and starch, strongly supporting the role of H_2_S in alleviating the senescence of tomato fruits. RT-qPCR result showed that the expression of beta-amylase encoding gene *BAM3* at late storage stage was inhibited by H_2_S treatment. Recently, it is reported that starch degradation was regulated by an ethylene responsive C2H2-type zinc finger transcription factor *AdDof3* in kiwifruit ripening and senescence (Zhang et al., 2018), thereby providing a cue to research the molecular mechanism of H_2_S in tomato fruit quality during storage.

Sugar content was the best criteria for evaluating the maturity of the fruit. The ratio of sugar/acid is an index which affects fruit flavor quality. In this study, H_2_S could maintain higher level of reducing sugar from 2 to 7 DAS and ascorbic acid contents than those of C_2_H_4_ treatment (Figures 5A,D). It was showed that reducing sugar has an obviously negative correlation with ascorbic acid (Figure 8A). Meanwhile, phenolic compounds are second metabolites in many fruits and well-known for their antioxidant potential, and their role in prevention of heart diseases, inflammation, and reducing the incidence of cancer and diabetes (Kaur and Kapoor, 2001). Previous report suggested a strong correlation between total antioxidant activity and total phenolic content (Macoris et al., 2012). The total phenols have a negative correlation with reducing sugar (Figure 8A). The results showed that H_2_S can effectively inhibit the decrease of reducing sugar and ascorbic acid, indicating that H_2_S contributed to improved antioxidant capacity to extend post-harvest storage period.

In our study, we demonstrated the indispensable role of H_2_S in delaying ripening and senescence of tomato fruits during the storage period. Our results showed H_2_S could maintained higher levels of metabolites, such as chlorophyll, starch, soluble protein and ascorbic acid. It implied that H_2_S might be an endogenous signal which regulated the ripening and senescence of post-harvest fruits by repression of the effect of ethylene.

## Data Availability Statement

The raw data supporting the conclusions of this article will be made available by the authors, without undue reservation, to any qualified researcher.

## Author Contributions

G-FY, CL, K-KS, K-DH, and HZ conceived and designed the experiments. G-FY, CL, K-KS, G-GH, Z-QH, PJ, and JT performed the experiments. G-FY, CL, FY, and L-YH analyzed the data. G-FY, CL, PJ, and K-DH wrote the manuscript. G-FY, K-DH, and HZ interpreted the data and revised the manuscript.

## Conflict of Interest

The authors declare that the research was conducted in the absence of any commercial or financial relationships that could be construed as a potential conflict of interest.

## Abbreviations

C_2_H_4_: ethylene
CHL: chlorophyllase
CO: carbon monoxide
DAS: day after storage
EDTA: ethylene diamine tetraacetic acid
FW: fresh weight
G–O: green–orange
G–Y: green–yellow
H_2_S: hydrogen sulfide
L-R: light red
NO: nitric oxide
O–R: orange–red
PCA: principal component analysis
PPH: pheophytinase
TA: titratable acid
UFGT: UDP glucose: flavonoid-3-*O*-glucosyltransferase.
