# Supplementary material for: Hydrogen Sulfide Maintained the Good Appearance and Nutrition in Post-harvest Tomato Fruits by Antagonizing the Effect of Ethylene
Source: Front Plant Sci. 2020 May 14;11:584. doi: 10.3389/fpls.2020.00584 (PMC7240128; doi:10.3389/fpls.2020.00584)
Supplement: Supplementary file 1 [file Table_1.DOCX]

Table S1. Primers used for the quantitative polymerase chain reaction.

| Gene name | Accession number | Primer sequences for qPCR (5'-3') |
| --- | --- | --- |
| *BAM3* | LOC101259175 | ACATGGCTCTATACCGGATTTC |
|  |  | GCCTAACTTCTGCGGGATTAT |
| *UFGT73* | LOC101257246 | CCGTGCTCTACGTTTGTCTT |
|  |  | ACCCATACGAAGGGTTGTTTAG |
| *UFGT5* | LOC101256157 | CCGGTTGGACCATTGATTAGA |
|  |  | CCACCACTCCCGAAAGATATG |
| *ERF003* | Solyc06g068360 | CTCGTCCACAACAACGATTTC |
|  |  | GTACCTAGCCATATTCTCGTCTTC |
| *DOF22* | NM_001328434.1 | GCAGTGTAGAGAAGGATGAAGG |
|  |  | TGGTCTCCCTCCTCAGATTTA |
| *WRKY51* | LOC101258361 | GCTCATCAAACCCTAACCCTAA |
|  |  | AATCGTCACTCGATCCATCATC |
| *SlTubulin* | Solyc08g006890 | TAGAGCCTGGTACGATGGATAG |
|  |  | CAACTCAGCGCCTTCAGTATAA |

The first line of primer is forward primer, the second line reward primer.
